# Deep inspiration breath-hold (DIBH) technique applied in right breast radiotherapy to minimize liver radiation

**DOI:** 10.1259/bjrcr.20150038

**Published:** 2015-05-11

**Authors:** L Rice, S Harris, M M L Green, P M Price

**Affiliations:** ^1^The Harley Street Clinic, London, UK; ^2^Department of Surgery and Cancer, Imperial College London, London, UK

## Abstract

A right-sided breast cancer patient (stage T1N0M0) was referred for post-surgical radiotherapy to minimize risk of local tumour recurrence. During the CT simulation and intensity-modulated radiotherapy planning process undertaken in free breathing, it was apparent that an unusually large volume of normal liver tissue (134 cc) was in the high-dose region of the tangential radiation field. This raised concern for risk of liver side effects and was considered suboptimal for this excellent prognosis patient. A deep inspiration breath-hold (DIBH) technique using three-dimensional (3D) surface monitoring—primarily developed and applied in left breast cancer to displace cardiac tissue from the target field—was investigated to determine potential benefit to optimize radiotherapy delivery. Resimulation of DIBH resulted in considerable displacement of the liver, reducing the volume of liver tissue in the target field by 63% (to 50 cc) and the mean liver dose by 46% (to 2.6 Gy). As the patient was deemed suitable for the DIBH technique, treatment was delivered according to the DIBH plan. A total of 40.05 Gy in 15 fractions was successfully delivered in the DIBH position using a technique that incorporated 3D body surface imaging with automated radiation beam hold-off when out of tolerance. Additional advantages were optimal set up without extensive immobilization and the elimination of respiratory motion. Acute mild skin erythema was the only side effect experienced—no liver sequalae were experienced by the patient up to 6 months after treatment. DIBH treatment may improve liver sparing in other similar right breast cancer patients.

## Clinical presentation

This 62-year-old female patient presented via the breast screening programme with a lesion in the upper outer quadrant of the right breast. The tumour was surgically removed via wide local excision and a sentinel lymph node biopsy was carried out. Histology showed a 12-mm grade I infiltrating tubular cancer with associated low-grade ductal carcinoma *in* *situ* and no vascular invasion. Surgical margins were clear and the sentinel node was not involved with the tumour. The tumour was staged as T1N0M0 and was determined to be oestrogen-receptor positive and HER2 negative. After surgery, the patient received adjuvant endocrine therapy and a course of radical radiotherapy was prescribed for the conserved right breast to reduce the risk of recurrence.

## Investigations/imaging findings

**Figure 1.**
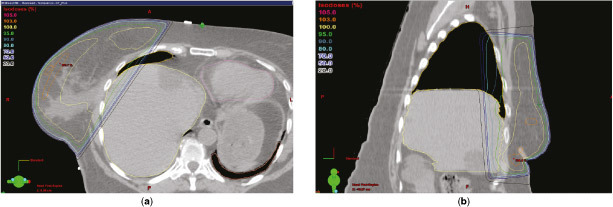
Free-breathing transverse (a) and sagittal (b) planning CT images from Eclipse™ planning system (Varian Medical Systems, Palo Alto, CA). The delineated liver (light yellow contour line), right lung (dark yellow contour line), left lung (orange contour line) and heart (pink contour line) are shown in relation to the target field. A large volume of liver is within the target field, including in the high 95% isodose region (bright green contour line).

**Figure 2.**
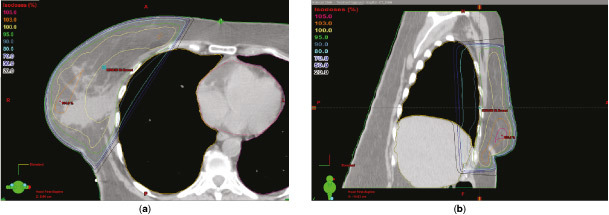
Deep inspiration breath-hold transverse (a) and sagittal (b) planning CT images from the Eclipse™ planning system (Varian Medical Systems, Palo Alto, CA). The liver (light yellow contour line) is displaced inferiorly and posteriorly away from the high 95% isodose treatment area (bright green contour line) as the right (dark yellow contour line) and left lung (magenta contour line) are fully inflated.

Radiotherapy planning using CT simulation was initially carried out in free breathing (FB) with the patient in the supine position on a Quest RT-4543 breastboard (Q Fix Systems, Avondale, PA). A GE Lightspeed RT 16 scanner (General Electric, Fairfield, CT) was used to generate spiral CT images with slice thickness of 2.5 mm. The CT data sets were transferred to the Eclipse™ planning system (Varian Medical Systems, Palo Alto, CA) through a Digital Imaging and Communications in Medicine network. A forward-planned intensity-modulated radiotherapy (IMRT) treatment plan was prepared by trained dosimetrists in conjunction with the treating clinician. A field-in-field approach was used, incorporating three medial and three lateral tangential beams, integrating a mix of 6 and 10 MV beams, to provide a homogenous dose distribution throughout the breast tissue to a prescribed total dose of 40.05 Gy in 15 fractions. Plans were produced according to International Commission on Radiation Units guidelines to achieve 95% isodose coverage to the clinical target volume (CTV), limiting the maximum isodose to 105% of the prescribed dose. The CTV encompassed the whole intact breast and chest wall, including the soft tissues of the deep pectoral fascia, within the back edge of tangential fields and clinical skin marks of established treatment fields using Radiation Therapy Oncology Group breast cancer contouring atlas definitions.^1^ The medial border was defined by the midline sternum–rib junction, and the lateral border as 1 cm below the breast plate or to the midaxillary line, excluding the latissimus dorsi muscle. The superior border was placed at the second intercostal space at the level of the angle of Louis and the inferior border was 1–2 cm below the extent of CT-apparent breast tissue. A planning target volume expansion was not applied. The heart, liver and both ipsilateral lung (iLung) and contralateral lung (cLung) were outlined and treated as organs at risk (OAR), applying stringent mandatory low-dose constraints to the heart and lungs [heart volume receiving >13 Gy (V_13 Gy_) <10%, iLung volume receiving >18 Gy (V_18 Gy_) <15%, cLung volume receiving >2.5 Gy (V_2.5 Gy_) <15%] as developed for the IMPORT high trial.^2^ The FB planning scan (Figure 1) showed an unusually large volume of normal liver located in the high-dose target area (134 cc). Given broad guidelines suggesting ≤5% radiation-induced liver disease (RILD) risk with a mean liver dose of ≤30–32 Gy,^3^ the doses to this partial liver volume were unlikely to have clinical relevance for the development of RILD in this case. However, the liver radiation associated with the FB plan was considered undesirable for this excellent prognosis patient, as well as being discordant with our aims to minimize all OAR radiation while ensuring good target coverage. Transient post-radiotherapy changes in liver function tests and radiographic reductions in liver density are also reported to be common where the liver is incidentally irradiated.^3^ A method to reduce the dose to the liver was therefore sought. Although heart and lung toxicity is widely addressed in the current literature,^4–9^ radiation-associated liver toxicity is recognized^3^ but not well studied in relation to breast radiotherapy. We found only one report on liver sparing in right-breast cancer—a planning study that concluded excellent liver sparing in right breast cancer using deep inspiration breath-hold (DIBH).^10^ A DIBH technique, using three-dimensional (3D) surface monitoring for automated beam-hold delivery, has been developed for use at our clinic to reduce incidental heart dose in suitable left breast cancer patients. The potential benefits and suitability of the patient for DIBH radiotherapy delivery technique were therefore investigated. The patient subsequently underwent a repeat radiotherapy planning CT simulation scan in the DIBH position (Figure 2). DIBH resulted in considerable displacement of the liver away from the high-dose target region, such that the volume of liver in the high-dose region was reduced by 63% to 50 cc. A full treatment plan was generated on the Eclipse planning system in exactly the same way as was the FB plan to compare the dosimetric statistics of the two plans (Table 1). All liver dose metrics were substantially reduced with the DIBH plan, with the volume of liver receiving ≥30 Gy (V_30 Gy_) reduced by 64%. Heart dose metrics showed negligible differences between the two plans. Some lung dose metrics generated by the planning system using dose–volume data showed increases with DIBH compared with FB. As lung mass typically decreases by approximately 40% in DIBH, it is acknowledged that dose–mass data may have aided the assessment of lung dose with variable inflation.^9^ However, in all cases, lung dose metrics remained below the low threshold safe limits imposed.^2^ As breathing motion is eliminated with DIBH technique, the set-up was anticipated to be optimal without the need for extensive immobilization (based on experience with other patients), and the patient fulfilled all eligibility criteria developed at our clinic for our DIBH radiotherapy delivery technique (Table 2), the DIBH treatment plan was judged to be overall more favourable and was therefore selected for treatment.

**Table 1.** Summary of OAR dosimetrics for FB and DIBH plans

OAR (data generated by Eclipse^−^ planning system)		FB	DIBH	Difference (%)
Liver	Volume within target field	133.98 cc	50.16 cc	−63.00
	Mean dose	4.8 Gy	2.6 Gy	−46.00
	Maximum dose	40.0 Gy	38.6 Gy	−3.5
	V_30 Gy_	6.9 cc	2.5 cc	−64.00
	V_20 Gy_	8.1 cc	3.4 cc	−58.00
	V_10 Gy_	9.6 cc	4.7 cc	−51.00
Heart	Mean dose	0.4 Gy	0.4 Gy	±0.00
	Maximum dose	2.1 Gy	2.2 Gy	+5.0
Right lung	Mean dose	5.1 Gy	7.0 Gy	+37.00
	Maximum dose	39.1 Gy	39.2 Gy	+0.26
	V_30 Gy_	5.9	10.4	+76.00
	V_20 Gy_	7.7	12.9	+67.00
	V_10 Gy_	10.4	17.1	+64.00
Left lung	Mean dose	0.02 Gy	0.04 Gy	+100.00
	Maximum dose	0.4 Gy	0.6 Gy	+50.00

DIBH, deep inspiration breath-hold; FB, free-breathing; OAR, organs at risk.

**Table 2.** Summary of screening criteria used for application of DIBH technique at Harley Street Clinic Radiotherapy Department

Potential benefit of DIBH method	• OAR in free-breath treatment field that is expected to be displaced by DIBH
No alternative method to improve planning dosimetry	• Multi-leaf collimation considered as unsuitable/inferior alternative
Adequate DIBH chest breathing reproducibility	• Patient instructed in 20 s DIBH and supervised through several practices • Patient able to follow breath-hold instructions • Regular rib cage rise and fall seen when anterior tattoo displacement measured on breastboard scale
No patient-specific factors that would compromise DIBH setup reproducibility	• Sufficient shoulder movement, comfortable lying flat, stable breast tissue etc.
Benefit of DIBH method confirmed	• Planning CT in DIBH shows OAR displacement

DIBH, deep inspiration breath-hold; OAR, organs at risk. Eclipse planning system manufactured by Varian Medical Systems , Palo Alto, CA

## Treatment

The patient was treated within 20 days according to the DIBH forward-IMRT treatment plan. Radiotherapy was delivered with a Trilogy™ Linac (Varian Medical Systems UK Ltd., Crawley, UK). During treatment, a 3D surface imaging system (AlignRT® Beam-Hold; VisionRT Ltd., London, UK) was used to achieve a stable, reproducible breath-hold position and track real-time patient motion in six degrees-of-freedom. The AlignRT system communicates directly with the Varian Linac to activate "Beam-Hold" when a patient's position falls out of tolerance with the planned CT scan. Video goggles were used to provide the patient with visual feedback and coaching for DIBH reproducibility and stability.^8^ Pre-treatment medial field verification image was performed daily using the PortalVision™ MV system (Varian Medical Systems, Palo Alto, CA) with images matched online using a tolerance of 0.5 cm.

## Outcome and follow-up

The patient completed all treatment fractions on the DIBH plan as scheduled, with no difficulties experienced. The only acute side effect experienced was mild erythema in the treatment area. No liver, lung or heart side effects were reported up to 6 months after treatment.

## Learning points

1. Adverse effects of breast cancer treatment have become increasingly important as survivorship has improved. Techniques to minimize dose to adjacent OAR are necessary to lower risks.
2. DIBH methods have been developed and applied primarily to reduce heart dose in left breast cancer because of the known risks of cardiac toxicity.^4,6–8^ During DIBH, the heart and upper abdominal organs are displaced away from the treatment field.
3. DIBH may be appropriate for some right breast cancer patients where high volume of liver tissue in the treatment field results in suboptimal protection of the organ from incidental radiation.^3,10^ To our knowledge, this is the first report of a gated DIBH technique applied to treat a right breast cancer patient specifically to reduce liver dose.
4. Suitability for DIBH should be judged on an individual patient basis. Risk of liver side effects may vary with co-morbidity (e.g. hepatitis/poor liver function) and concurrent chemotherapy,^3^ individual anatomical differences contribute to variable proportional benefit of DIBH and patient suitability and compliance can affect DIBH technique reproducibility.
5. The additional advantages of the DIBH technique used are optimal set-up without the need for extensive immobilization and the elimination of breathing motion, which may improve optimization of the dose distribution calculation and the accuracy of RT delivery. The associated challenges of the technique include: the need for patient selection and an additional CT scan, the financial and space implications for extra equipment required and the additional time required for staff training, patient coaching and daily quality assurance.
